# Brain metabolic alterations herald falls in patients with Parkinson’s disease

**DOI:** 10.1002/acn3.51013

**Published:** 2020-03-11

**Authors:** Ioannis U. Isaias, Joachim Brumberg, Nicoló G. Pozzi, Chiara Palmisano, Andrea Canessa, Giorgio Marotta, Jens Volkmann, Gianni Pezzoli

**Affiliations:** ^1^ Department of Neurology University Hospital and Julius Maximilian University of Würzburg Würzburg Germany; ^2^ Department of Nuclear Medicine University Hospital Würzburg Würzburg Germany; ^3^ Fondazione Europea di Ricerca Biomedica (FERB Onlus) Cernusco s/N (Milano) Italy; ^4^ Department of Nuclear Medicine Fondazione IRCCS Ca' Granda – Ospedale Maggiore Policlinico Milano Italy; ^5^ Centro Parkinson ASST G. Pini‐CTO Milano Italy

## Abstract

Pathophysiological understanding of gait and balance disorders in Parkinson’s disease is insufficient and late recognition of fall risk limits efficacious follow‐up to prevent or delay falls. We show a distinctive reduction of glucose metabolism in the left posterior parietal cortex, with increased metabolic activity in the cerebellum, in parkinsonian patients 6–8 months before their first fall episode. Falls in Parkinson’s disease may arise from altered cortical processing of body spatial orientation, possibly predicted by abnormal cortical metabolism.

## Introduction

Falls and fall‐related injuries are a major cause of disability in subjects with Parkinson’s disease (PD). Fall rates in PD range from 35 to 90% and increase during the disease course.^1^ Despite detailed testing of gait and balance, the specific factors that are critical to fall prediction and prevention in PD remain elusive^2^ and the best single variable to predict falls is two or more falls in the previous year (odds ratio 1.5 or higher).^3^, ^4^, ^5^ Late recognition of patients at risk of falls prevents their timely treatment and is mainly related to the unclear pathophysiology of balance dysfunction in PD. While nigrostriatal dopaminergic denervation does not differ between the PD patients with and without falls,^6^ functional brain imaging studies has recently showed a direct involvement of cortical areas in PD fallers.^6^, ^7^ Brain metabolic imaging with 2‐deoxy‐2‐[18F]fluoro‐D‐glucose ([18F]DG) and positron emission tomography (PET) can reliably identify the symptom‐specific brain metabolic changes for tracking the progression of neurodegenerative processes and their response to treatment.^8^ In this study, we investigated the brain metabolic alterations in PD patients at high risk of fall prior to the occurrence of the first fall.

## Patients and Methods

### Study subjects

We retrospectively evaluated the clinical records and molecular imaging findings of over 200 patients with a diagnosis of idiopathic PD who underwent a PET with [18F]DG between 2012 and 2017 as part of the workup for potential deep brain stimulation candidates or other research studies. We identified one group of 11 right‐handed patients (Fallers) who experienced their very first fall episode between 6 and 8 months after the execution of the [18F]DG PET. A time period shorter than 6 months would have reduced the predictive value of our findings and a longer time would have introduced several confounding factors (e.g. therapy changes, comorbidities, etc.). A fall was defined as an event of unintentionally coming to rest on the ground or lower level. The dopaminergic therapy was unchanged between the [18F]DG PET and the fall episode. We then selected a second group of 19 right‐handed patients (Non‐fallers) with similar demographic and clinical characteristics who never experienced any fall episode (up to 2‐year follow‐up) and 12 right‐handed healthy subjects (HC). Exclusion criteria were dementia (i.e. Mini‐Mental State Examination score < 25), clinically relevant depression (Beck Depression Inventory score > 8), significant comorbidities (e.g. visual disturbances, cardiovascular diseases including symptomatic postural hypotension, etc.), and abnormal structural MRI. PD patients with freezing of gait (including start hesitation) were not included in this study. The local Ethics Committee approved the study and informed consent was obtained from all participants.

### Imaging acquisition and analysis

PET acquisitions were performed as previously described.^9^ In brief, patients fasted overnight and stayed in resting conditions in a dimly lit and quiet room for 30 min between the injection of [18F]DG and PET acquisition. All scans were performed in the morning, approximately 2 h after the intake of the morning dose of antiparkinson medications. Iterative data reconstruction and CT‐based attenuation correction was applied. Data were analyzed using Statistical Parametric Mapping (SPM8). Scans were spatially normalized to a PET template in the standardized Montreal Neurological Institute space and then smoothed. Voxel‐by‐voxel comparison between the three groups (i.e. Fallers, Non‐fallers, and HC) have been explored by performing a one‐way analysis of variance (ANOVA), with age and disease duration as covariate. Then, inference on brain glucose metabolism differences between groups was made using double‐sided *t*‐tests (*P* < 0.001, cluster Family‐Wise Error corrected with an extended threshold of at least 150 contiguous voxels). We additionally performed a post hoc volume‐of‐interest (VOI) analysis using a spherical VOI (4 mm radius) centered on the peak voxel of significant clusters in SPM analysis and calculated the standardized uptake value ratio (SUVR) (i.e. mean count per voxel VOI/mean count per voxel global brain) of [18F]DG uptake within the SPM predefined brain areas.

### Statistical analysis

Statistical analyses were performed with JMP14. Gender distribution was investigated with Pearson’s chi‐square test. The normal distribution of the data were tested with the Shapiro–Wilk test and the equality of variances with the Levene’s test. Groups were compared with the Student t‐test or ANOVA or the Wilcoxon test, with post hoc analyses (i.e. the Tukey‐Kramer HSD test), when appropriate (Table). A multivariate logistic regression analysis was performed to identify which VOI measurement could independently predict a fall event (i.e. Fallers). We then performed an ANCOVA to establish the influence of age, disease duration, UPDRS‐III score, and Levodopa Equivalent Daily Dose (LEDD) as covariates on the prediction of VOI measurements by group (Fallers and Non‐fallers).

## Results

**Figure 1:**
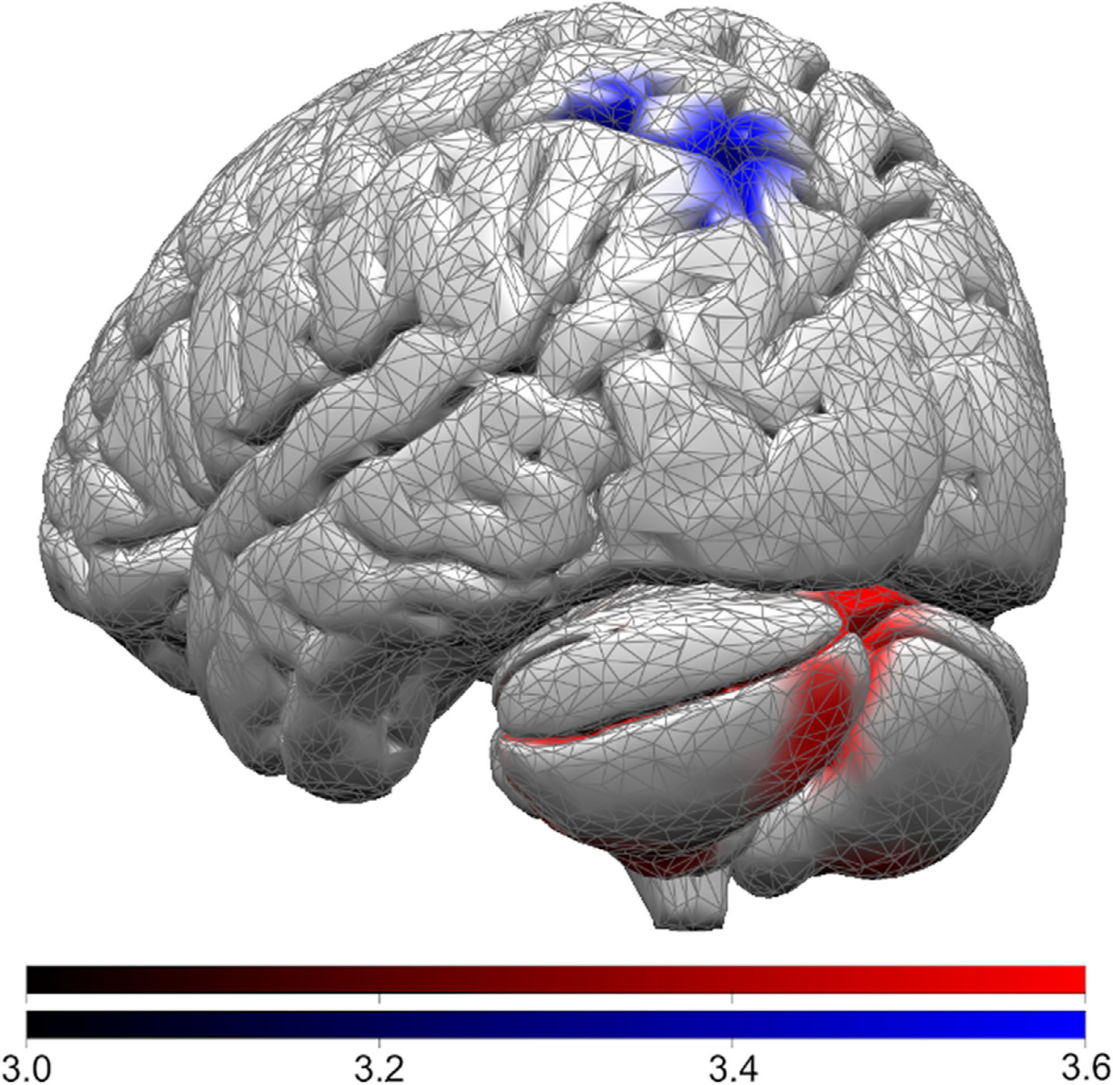
Brain areas with enhanced (red) or reduced (blue) glucose metabolism in Fallers (compared with non‐Fallers). The color bars indicate corresponding *t*‐values.

Fallers showed distinctive hypometabolism in the left parietal cortex (precuneus and inf. and sup. parietal lobule; cluster peak coordinates: *x* = −26, *y* = −77, *z* = 40; k = 305; p_FWEcorr_ = 0.04; Z‐score = 4.02) and increased bilateral cerebellar glucose consumption (left cerebellum post. lobe and tuber of vermis; cluster peak coordinates: *x* = −1, *y* = −70, *z* = 444; k = 444; p_FWEcorr_ = 0.009; Z‐score = 0.009 and right cerebellum post. and ant. lobe; cluster peak coordinates: *x* = 25, *y* = −44, *z* = 357; k = 357; p_FWEcorr_ = 0.023; *Z*‐score = 0.023) (Fig. 1 and Table 1). ANOVA analysis with SPM and VOI revealed that glucose metabolism significantly differed between the three groups in the left parietal cortex and bilateral cerebellar lobi and post hoc two‐sample t‐tests showed that Fallers versus Non‐fallers or versus HC showed hypometabolism in the left inferior and superior parietal lobules and increased bilateral cerebellar glucose consumption. Lastly, in our study population the [18F]DG uptake value of the left parietal cortex was the single measurement to independently predict future falls (log‐likelihood chi‐square test, FDR *P* = 0.001). The different [18F]DG uptake between Fallers and Non‐fallers cohort in the left parietal area was not influenced by demographic or clinical variables [*F*(5,24)=14.49, FDR *P* = 0.004 and SPM analysis].

**Table 1:** Demographic and clinical characteristics at the time of FDG PET and VOI measurements.

	Fallers	Non‐fallers	HC	*P* value Fallers vs. Non‐fallers	*P* value Fallers vs. HC	*P* value Non‐fallers vs. HC
Sex (F/M)	3/8	6/13	5/7	0.804^1^	0.469^1^	0.568^1^
Age (years)	63.64 ± 8.62	62.63 ± 9.04	61.75 ± 14.28	0.967^2^	0.906^2^	0.973^2^
Disease duration (years)	7.09 ± 4.18	8.16 ± 4.50	–	0.462^3^	–	–
UPDRS‐III (score 0 to 108)	34.64 ± 14.18	31.52 ± 12.87	–	0.546^3^	–	–
LEDD (mg)	840.0 ± 386.11	783.16 ± 311.24	–	0.662^4^	–	–
Left parietal lobe (SUVR)	1.03 ± 0.10	1.20 ± 0.10	1.16 ± 0.12	<0.001^2^	0.016^2^	0.679^2^
Left cerebellum, post. lobe, tuber of vermis (SUVR)	1.16 ± 0.08	1.05 ± 0.11	1.05 ± 0.07	0.007^2^	0.013^2^	0.994^2^
Right cerebellum, ant. lobe (SUVR)	1.20 ± 0.07	1.11 ± 0.07	1.11 ± 0.09	0.010^2^	0.018^2^	0.998^2^

Data are presented as mean ± standard deviation and corresponding p values (^1^Pearson’s chi‐square test, ^2^ANOVA and Tukey‐Kramer HSD test, ^3^Wilcoxon test, ^4^Student *t*‐test).

## Discussion

The posterior parietal cortex has been proposed as the sensorimotor interface responsible for the integration and timing of movement intentions with ongoing movements. While the right posterior parietal cortex may be relevant for visuospatial and covert‐orienting attention, the left posterior parietal cortex is responsible for matching between the anticipated and actual sensorimotor consequences.^10^, ^11^ The assignment of balance disorders and falls in PD to the left hemisphere can be made only tentatively on the basis of the present results. In our study, the selective impairment of the left parietal cortex might be related to the preferential role of this brain area in coding the spatial relationships between the discrete body parts.^12^, ^13^, ^14^, ^15^ Alterations in this brain area would impair the predictability of body orientation and the maintenance of certain relative positions of the body segments, thus directly impairing the balance control.^16^ A predominant involvement of the left parietal cortex in gait was also shown in other brain imaging studies with resting state^7^ or imaged locomotion protocols.^17^ A hypofunctioning left parietal cortex would also lead to downregulation of the activity in the primary motor cortex and supplementary motor area,^18^ delaying the adaptation of gait to environmental needs, and additionally increasing the risk of falls in parkinsonian patients.

Reactive and predictive sensorimotor adjustments are assumed to be ruled by internal models located in the cerebellum.^19^, ^20^ In this context, the posterior parietal regions would play key roles in sending signals representing intended motion for a proper prediction of sensory consequences of movement.^19^ The increased cerebellar activity in Fallers can be, therefore, a compensatory attempt for poor adaptability to motor patterns due to an impaired parietal cortex signaling.^20^ More in general, there is increasing evidence of compensatory cerebellar activation in parkinsonian patients,^21^ but the complex interplay between cerebellum and cortical areas following basal ganglia derangements has yet to be elucidated.^21^ Interestingly, Zhang and coll.^7^ failed to describe increased cerebellar activity, possibly suggesting exhaustion of such a compensatory activity when parkinsonian patients already manifest postural instability.

Our study has some limitations. First, this retrospective study can only reflect the temporary profile of PD falls, and further follow‐up studies are needed to assess the evolution of the risk of falling over time. Second, given the relatively small sample size, future longitudinal studies are required to define a specific pattern for diagnostic and predictive purposes at a single subject level. Third, since all patients did not refer balance disturbances or previous falls at the time of the clinical evaluation or [18F]DG PET study, they were not investigated with dedicated scales such as the Tinetti Test, the Berg Balance Scale or the Timed up, and go test. Still, these clinical scales have not been consistently reported as independent predictors of future falls in non‐faller PD patients.^4^, ^22^ Lastly, all patients performed the [18F]DG PET study in medication on state, but the brain metabolic differences we showed in this study cannot be related to an acute effect of dopaminergic drugs.^23^ Despite these limitations, for the first time we have been able to describe the brain metabolic alterations in PD patients at high risk of fall before the very first fall episode. This information can help monitoring patients and planning therapeutic and preventing interventions (e.g. physical therapy).^24^ Our findings further suggest the hypothesis that some PD‐related symptoms, in particular gait and postural disorders, might involve extra‐striatal areas and are not dopamine‐dependent,^25^ but instead represent a direct expression of network derangements and compensatory attempts or their failure.^26^

## Conflict of Interest

The authors declare that they have no conflict of interest.
